# A Cognitive Systems Engineering Approach Using Unsupervised Fuzzy C-Means Technique, Exploratory Factor Analysis and Network Analysis—A Preliminary Statistical Investigation of the Bean Counter Profiling Scale Robustness

**DOI:** 10.3390/ijerph191912821

**Published:** 2022-10-06

**Authors:** Dana Rad, Lavinia Denisia Cuc, Ramona Lile, Valentina E. Balas, Cornel Barna, Mioara Florina Pantea, Graziella Corina Bâtcă-Dumitru, Silviu Gabriel Szentesi, Gavril Rad

**Affiliations:** 1 Center of Research Development and Innovation in Psychology, Faculty of Educational Sciences Psychology and Social Sciences, Aurel Vlaicu University of Arad, 310096 Arad, Romania; 2 Faculty of Economics, Aurel Vlaicu University of Arad, 310096 Arad, Romania; 3 Faculty of Engineering, Aurel Vlaicu University of Arad, 310096 Arad, Romania; 4 Faculty of Exact Sciences, Aurel Vlaicu University of Arad, 310096 Arad, Romania; 5 Faculty of Accounting and Management Informatics, Department of Accounting and Audit, Bucharest University of Economic Studies, 010374 Bucharest, Romania

**Keywords:** bean counter, cognitive systems engineering, unsupervised learning, fuzzy c-means, exploratory factor analysis, network analysis, scale statistical architecture

## Abstract

A bean counter is defined as an accountant or economist who makes financial decisions for a company or government, especially someone who wants to severely limit the amount of money spent. The rise of the bean counter in both public and private companies has motivated us to develop a Bean Counter Profiling Scale in order to further depict this personality typology in real organizational contexts. Since there are no scales to measure such traits in personnel, we have followed the methodological steps for elaborating the scale’s items from the available qualitative literature and further employed a cognitive systems engineering approach based on statistical architecture, employing cluster, factor and items network analysis to statistically depict the best mathematical design of the scale. The statistical architecture will further employ a hierarchical clustering analysis using the unsupervised fuzzy c-means technique, an exploratory factor analysis and items network analysis technique. The network analysis which employs the use of networks and graph theory is used to depict relations among items and to analyze the structures that emerge from the recurrence of these relations. During this preliminary investigation, all statistical techniques employed yielded a six-element structural architecture of the 68 items of the Bean Counter Profiling Scale. This research represents one of the first scale validation studies employing the fuzzy c-means technique along with a factor analysis comparative design.

## 1. Introduction

A bean counter, according to a widely recognized definition, is a person, often an accountant or bureaucrat, who is regarded to place an undue focus on regulating expenditure and budgets, a person concerned in business or government financial choices, particularly one who is hesitant to spend money.

The term “bean counter”, also referred to as “bean keeper”, “number cruncher” [1], “ledger attendant”, “money guard” [2], or “corporate cop” [3,4,5,6], evokes an image of an accountant spending the whole day hunched over a calculator, trying to save expenses for the company on some form of expenditure or creating spreadsheets loaded with information to back up management’s decision to reduce staff or programs [7].

Although other financial controllers may also fulfill the definition, the word bean counter is generally used negatively to imply an overly zealous or meticulous accountant. While an accountant may be requested to conduct a detailed inventory of his or her firm’s assets, only a bean counter would count the amount of beans in the corporate kitchen’s storage. Such a person may also examine each department’s budget to identify any possible waste, no matter how minor or unimportant it appears to be. Project managers frequently fear this sort of accountant, as budget cuts and lengthy audits might follow soon behind. Bean counters were formerly financial comptrollers and accountants who took an unusual interest in even the tiniest elements of a company’s finances. Originally, practically everybody working in accounting professions was described informally in this way, with little to no negative connotation attached. Indeed, many professional accountants and comptrollers proudly use this appellation. However, in recent years, the term has not been favorable to accountants. A growing percentage of people blame financial experts for the elimination or termination of initiatives due to budgetary constraints. A bean counter is generally the first to discover when a cost over-run or budget limit is surpassed. When it comes to beans, an accountant needs to know when to declare a scarcity and when to learn how to survive without them.

Although significant research has been conducted into the image of accountants, notably in the media and popular films, existing publications have mostly studied how people see accountants and how accountants are commonly depicted. When appealing to accountants, the publication, on the other hand, lays greater emphasis on the creation of their image [8].

Identity-building questions provide insight into the extent to which public stereotypes fit professional accountants’ own definitions as well as the impact these may have on present and future career plans. Including findings can serve as the foundation for a variety of profession policy challenges such as recruiting, retention, training, and professional growth [9].

Businesses must increasingly use extensive data mining and analytics to compete in this information age. This requirement has created a once-in-a-lifetime opportunity for accountants to play a more strategic role in their firm, together with their reputation for comprehending and creating high-quality data and the growing accessibility of analytics tools [10].

The image of a bean counting controller has permeated theoretical literature, popular culture, and programmatic, marketed management goals. The bean counter controller has been represented as a formal agent who values precision and precise financial analysis, usually in functional and social isolation. The bean counter controller is an autonomous but narrow technical instrumentalist, a quiet and sometimes distant processor of management accounting information, someone who avoids organizational connection, initiative, and wider participation, preferring to operate as a watchdog [11].

This study places the concept of the bean counter controller under conceptual statistical examination in the East-European countries context. Foremost, the study seeks to update research on measuring established behaviors of bean counters by elaborating a Bean Counter Profiling Scale, based on previous qualitative research. A number of items were developed according to methodology [12]. Since this preliminary research paper is focused solely on the statistical approach of the scale design, we will not further describe the in-depth procedure for items generation. We have opted to publish this preliminary investigation for an in-depth description of the original statistical procedure approach to establish the robust factorial structure of the BCP scale (factor, cluster and network analysis) strictly from the mathematical point of view. This enlarged approach is needed, since there are no validated scales of bean counter profiling reported in the scientific literature and no reported theoretical frameworks that could offer a binding to personality and profiling research, even if much emphasis is put on controlling finances and personnel training in this regard. We consider that a robust Bean Counter Profiling Scale will further develop the accountant profession and research.

Thus, the need for such a scale together with the need for a novel validation technique which harnesses the power of machine learning from the cognitive systems engineering perspective has further motivated us to apply several novel and traditional statistical procedures for uncovering clusters and factors among well-established bean counter behaviors and attitudes, which we have earlier transformed into 68 items, since there are no theoretical approaches to define a bean counter so far. To guarantee that the final product or service is both effective and reliable, cognitive systems engineering is a professional field that use systematic methods of cognitive analysis and cognitive design.

We train a machine learning model to discover clusters in our data set in this research. A clustering process aims to uncover structures in data. To do so, the algorithm must determine the number of structures/groups in the data and how the characteristics are distributed within each group. Because an unsupervised learning algorithm simply accepts features as input, no proper labels are required. The purpose of unsupervised learning is to find clusters that indicate similarities across characteristics, allowing us to illustrate major patterns in describing a bean counter profile from the scientific literature. This research represents one of the first scale validation studies employing the fuzzy c-means technique along with a factor analysis comparative design.

Recent research [13,14,15,16,17,18,19,20] has demonstrated the superiority of fuzzy c-means over the classical k clustering algorithm and factor analysis in optimization problematics.

Since the focus of this paper is to provide detailed statistical argumentation for the factorial model chosen for our Bean Counter Profiling Scale, we are reporting this methodology as a preliminary investigation. In the validation study, we will focus on qualitative interpretation of the factorial structure in terms of items selected and the overall statistical descriptors of the Bean Counter Profiling Scale.

## 2. Statistical Architecture

The process of changing raw data into features that better describe the underlying problem to predictive models, resulting in enhanced model accuracy on unseen data, is known as feature engineering [21].

We will further employ the statistical architecture term to describe the cognitive systems engineering approach to validating our Bean Counter Profiling Scale; namely, we will further adopt traditional and innovative statistical procedures based on machine learning techniques and a structural visualization technique. The statistical architecture will further employ the cluster, factor and items network analysis to statistically depict the best mathematical design of the scale.

A typical cluster analysis includes three steps: dimension reduction, cluster identification, and result evaluation. Data sets are often made up of many observations with a variety of characteristics. Each element adds another dimension to the data, making it difficult to show the data fully in a two-dimensional graphic. Furthermore, clustering algorithms are afflicted by the propensity to perform badly if a large number of characteristics are added [22].

Dimensionality reduction methods that project multiple characteristics into a low-dimensional space can solve both of these difficulties. The next stage is to identify subgroups, for which there are several methods. Finding clusters and assigning individual observations to those clusters is a feature shared by all approaches. Distance to a cluster centroid (k), dissimilarity and connectivity between groups of data (agglomerative hierarchical clustering), or density can all be used (hierarchical density-based clustering). Not every approach assigns every observation to a cluster. There are other "fuzzy" algorithms (c) that estimate how much each observation corresponds with each cluster. Latent class analysis and latent profile analysis (for continuous data) are two instances of this (for binary data).

Mixture models conceptually resemble fuzzy clustering in that they evaluate the probability that an observation belongs to a cluster. The findings of the cluster analysis must be quantified as the last stage.

Two distinct statistical techniques used in data analytics are cluster analysis and factor analysis, which are frequently used in disciplines such as behavioral sciences. Both analytical processes have such names because they allow users to divide data into clusters or components. The fact that both of these techniques are essentially identical perplexes most newly established data analysts. While these two tactics appear to be identical on the surface, they differ in several ways, including their applications and goals.

A key contrast between cluster analysis and factor analysis is that they serve different purposes. The basic goal of factor analysis is to understand how the variables relate to one another and to explain the connection in a data set. Contrarily, the goal of cluster analysis is to address the variation in distinct data sets. To put it another way, factor analysis is intended to make things simpler, whereas cluster analysis aids in classification. By combining several variables into a smaller group of factors, factor analysis minimizes the number of variables. By dividing the data into fewer groups, cluster analysis lowers the amount of observations. As with factor analysis, there is no separation between independent and dependent variables in cluster analysis.

Both factor analysis and cluster analysis use unsupervised learning to segment data. Many recent researchers in this field think that factor analysis and cluster analysis are interchangeable terms. Despite appearing identical, they are not the same. Cluster analysis and factor analysis have different objectives. The objective is to divide the observations into distinct, homogeneous groups. On the other hand, component analysis explains the homogeneity of the variables because of value similarity. Complexity is another distinction between cluster and factor analysis. The size of the data clearly affects how the analysis is conducted. Cluster analysis becomes computationally challenging for huge data sets. The solution to a problem is the same in factor analysis as it is in cluster analysis, but factor analysis provides the researcher with a more complete answer.

Finally, the visual representation of data allows for the quantification of qualitative codes via network analysis as well as the investigation of network indices’ relationships with other quantitative factors via standard statistical processes [23].

Network analysis may be regarded of as a set of methodologies with a shared methodological viewpoint that allows researchers to depict interactions among actors and investigate the social structures that emerge as a result of the recurrence of these ties. The basic idea is that examining the links between objects leads to more accurate interpretations of the interaction processes. This analysis is performed by compiling relational data into a matrix.

For a better comprehension of the statistical architecture design, we will further present in the Table 1, the main advantages of each factor–cluster–network approach used in the present pre-validation study that can help researchers develop new profiling scales in selecting their methodological approach.

The study of Hofstetter and collaborators [24] provided a clear delineation between factor analysis and cluster analysis. A comprehensive comparison between factor analysis and network analysis was provided by Lee and collaborators [25], and the study of Ferligoj and collaborators [26] offered a comparative framework between cluster analysis and network analysis; still, no study has provided a comparative analysis between factor, cluster and network analysis so far.

### 2.1. Cluster Analysis

Clustering is a technique for identifying segments or groupings in a data set. Each data point is clustered or grouped to a single cluster in hard clustering. Each data point may or may not totally belong to a cluster. K-Means hard clustering is a clustering method. It divides the data into k-clusters. Instead of placing each data point into a separate cluster, soft clustering assigns a likelihood for that point to be in that cluster. Each data point in soft clustering or fuzzy clustering can belong to numerous groups, as can its probability score or likelihood. The fuzzy c-means clustering (FCM) technique is a popular soft clustering algorithm.

The soft clustering technique known as fuzzy c-means clustering assigns a likelihood or probability score to each data point, indicating whether or not it belongs to that cluster. Fuzzy c-means clustering is a better method than the k-means technique. The fuzzy c-means algorithm permits data points to potentially belong to many clusters, in contrast to the k-means method, which only allows data points to belong to one cluster. Fuzzy c-means clustering results are noticeably better for overlapping data sets.

The fuzzy iterative self-organizing data analysis technique (ISODATA), also known as fuzzy c-means clustering (FCM), is a clustering algorithm that uses membership degrees to determine how much each data point belongs to a certain cluster. Fuzzy c-means (FCM) is a data-clustering technique that splits a data set into N clusters, with various degrees of membership for each data point in the data set in each cluster. Fuzzy c-means is a well-known fuzzy clustering technique. One may create a fuzzy partitioning from data using an unsupervised clustering method. The method is dependent on a parameter m that denotes the level of fuzziness in the result.

K-means clustering divides the whole data set into k clusters, with each data point belonging to just one cluster. Fuzzy c-means generates k clusters and then assigns each data point to one of them; however, there is a factor that determines how strongly the data belong to that cluster. The main advantage of fuzzy c-means clustering is that it allows gradual memberships of data points to clusters measured as degrees in [0,1]. This gives the flexibility to express that data points can belong to more than one cluster.

### 2.2. Factor Analysis

Factor analysis is a statistical data reduction and analysis approach used to explain relationships between different outcomes as the product of one or more underlying causes or factors. The approach uses data reduction to try to express a group of variables with a reduced number. Factor analysis seeks to identify unexplained variables that impact covariation across numerous data. These variables indicate basic notions that are insufficiently measured by a single variable. Factor analysis is especially common in survey research, where each question’s replies indicate an outcome. Because many questions are frequently connected, underlying causes may have an impact on the subject’s replies. Because the goal of factor analysis is to find underlying factors that explain correlations across various outcomes, the variables analyzed must be at least partially associated; otherwise, factor analysis is ineffective.

Factor analysis is an exploratory investigation that aids in the classification of comparable variables into dimensions. It may be used to make the observations less dimensional in order to make the data easier to understand. For factor analysis, there are several rotation techniques. Data reduction typically involves the use of factor analysis. The exploratory technique is used when you do not already know the structures or dimensions of a group of variables. The confirmatory approach is used when one wants to test a specific hypothesis about the structures or dimensions of a set of variables.

To screen scale items and discover groupings that will further enable us to choose the most representative items, this study uses an exploratory factor analysis. The most popular technique is principal component analysis, where factor weights are calculated to extract the greatest variation feasible until there is no more useful variance.

### 2.3. Network Analysis

As a collection of approaches with a common methodological stance, network analysis enables researchers to visualize operator interactions and explore the social structures that develop as a result of the recurrence of these linkages. The main principle is that understanding the relationships between items enables us to analyze interaction processes more precisely. Relational data are compiled into a matrix and then used for this analysis. The idea of a social network becomes a useful analytical tool that makes use of the mathematical language of graph theory and the linear presumptions of matrix algebra when items are represented as nodes and their relationships are represented as lines linking pairs of nodes.

Network analysis, in comparison to most other quantitative social science topics, has paid minimal attention to statistical problems. Most techniques and measures examine the structure of specific data sets without accounting for sample variation, measurement error, or other unknown variables. Such difficulties are complex because of the dependency inherent in network data, but they are gaining traction.

Psychometric network analysis is a novel method for investigating the structure of psychological items. The majority of the psychometric network literature so far has been on measuring constructs (for example, dimensional structure); nevertheless, this is only one aspect of psychometrics. In this study, we looked at whether network analysis may be used as a tool for scale development.

## 3. Materials and Methods

### 3.1. Participants

In total, data were collected from 433 participants, of which (83) 19% identified as male gender and (351) 81% identified as feminine gender. In terms of age, our research sample covered the segment between 21 and 70 years old, with an average mean of 43 years old. As for previous work experience, our sample respondents registered values between 1 and 52 years of professional experience in financial departments, with an average mean of 20 years.

In terms of the sociocultural context of the present study, over the past few decades, there have been several significant changes to the sociocultural environment in which people live [27]. According to life span psychological theory [28], both ontogenetic and historical forces influence how an individual develops. From this perspective, results of the present study might be extrapolated for the East-European block countries.

### 3.2. Materials

The 68 self-reported items of the Bean Counter Profiling Scale (BCPS) were extracted from relevant existent qualitative literature [29,30,31,32,33,34,35,36,37,38,39,40,41,42,43,44,45], based on the methodology described by [12]. As the purpose of this preliminary investigation is not describing in detail the methodology used in the BCPS items development, we will further focus on the statistical properties of the scale. The BCPS 68 items scale reported an overall 0.947 Cronbach’s α coefficient, which demonstrates a very high reliability statistic.

An example item is “Draw management’s attention to the financial implications of the company’s actions”. Responses were registered on a Likert-type scale, ranging from 1 = “This statement does not characterize me at all”, to 5 = “This statement totally characterizes me”.

### 3.3. Procedure

The online version of the questionnaire was sent by email to previously targeted individuals with solid background in financial departments from Romania. In total, 433 randomized selected participants completed the 68 items version of the BCPS during June and July 2022. A standard protocol for administering the questionnaire was used [46].

## 4. Results

### 4.1. Fuzzy C-Means Clustering Results

Fuzzy c-means clustering is a fuzzy partitioning approach that outputs the degree of connection between each observation and each cluster. This allows data observations to be partially allocated to several clusters and provides substantial confidence in cluster membership. Apart from the soft approach, the technique of fuzzy c-means clustering is quite similar to that of k-means clustering.

Each data point is clustered or assigned to any one cluster in the k-means clustering algorithm for hard clustering [47]. Each data point may fully or partially belong to a cluster. Instead of grouping every data point into its own cluster, soft clustering assigns each point a likelihood of being in that cluster. Any data point can adhere to many clusters in soft clustering or fuzzy clustering, which is coupled with a probability score or likelihood [48]. The fuzzy c-means clustering (FCM) algorithm is one of the most used soft clustering methods [49]. Fuzzy c-means clustering has been successfully used in previous psychological clustering research to examine and find the music features that can calm individuals and which form the most relaxing music for therapeutic use [50], to analyze missing data [51], to analyze employee branding typology [52] and to investigate language processing [53].

We have processed our data using R-packages: cluster, e1017, and Rtsne [54,55,56,57,58,59,60].

The algorithm was set to run for a maximum of 25 iterations, with a maximum of two fuzziness parameters and 10 clusters. Measures of model performance that take model complexity into consideration are provided by the Akaike information criterion (AIC) and the Bayesian information criterion (BIC). In AIC and BIC, a condition that measures how well the model fits the data is combined with a term that penalizes the model proportionately to the number of parameters. A statistic used to assess the efficacy of a clustering method is the silhouette coefficient, which is often known as the silhouette score. Its value ranges from [−1, 1], where 1 indicates that the clusters are clearly distinct from one another and are spaced widely away from one another. Values near 0 denote overlapping clusters. The worst value is −1; the negative scores mean that data belonging to clusters may be wrong or incorrect. The following formula may be used to calculate the silhouette score:

$$\mathrm{Score}\;\mathrm{for}\;\mathrm{the}\;\mathrm{silhouette}\;=\;\frac{\left(p-q\right)\;}{\max\;\left(p,q\right)}$$

where

p = average intra-cluster distance, i.e., the average distance between each point within a cluster, and

q = average inter-cluster distance, i.e., the average distance between all clusters.

The model optimized with respect to the BIC value calculated six clusters (N = 433), with an R² coefficient of 0.242, indicating the amount of variance explained by the model, an AIC of 25,407.550, a BIC of 27,067.470 and a silhouette of 0.010. The between sum of squares of the six-cluster model is 7870.66 and the total sum of squares of the six-cluster model is 32,462.21.

The size of each cluster as seen in Table 2 is 11 observations for Cluster 1, 10 observations for Cluster 2, 262 observations for Cluster 3, 3 observations for Cluster 4, 130 observations for Cluster 5 and 16 observations for Cluster 6. As for the explained proportion of within-cluster heterogeneity, our model has obtained the following values: 0.017 for Cluster 1, 0.027 for Cluster 2, 0.708 for Cluster 3, 0.006 for Cluster 4, 0.192 for Cluster 5 and 0.050 for Cluster 6. The between sum of squares obtained for Cluster 1 is 423.931, for Cluster 2 is 657.042, for Cluster 3 is 17,400.350, for Cluster 4 is 157.315, for Cluster 5 is 4713.965 and for Cluster 6 is 1238.947.

The silhouette scores for Cluster 1 is 0.013, for Cluster 2 is 0.051, for Cluster 3 is -0.027, for Cluster 4 is 0.005, for Cluster 5 is 0.101 and for Cluster 6 is −0.087. As seen, four clusters obtained reasonable values, presenting overlapping information, which is expected in the case of our 68 items referring all to the bean counter profile, except for Cluster 3 and Cluster 6, which represent a bad pick for the given data due to the presence of clusters with below average silhouette scores (Table 2).

In terms of evaluation metrics, the maximum cluster diameter in Euclidean distance obtained was 24.182, the minimum cluster separation in Euclidean distance obtained was 4.040, Pearson’s γ value was 0.195: namely, the correlation between distances and a 0-1-vector where 0 means the same cluster, and 1 means different clusters. The Dunn index: minimum separation/maximum diameter value obtained was 0.167, the entropy of the distribution of cluster memberships was 1.002 and the Calinski–Harabasz index, the variance ratio criterion of the cluster memberships, was 16.341.

**Figure 1:**
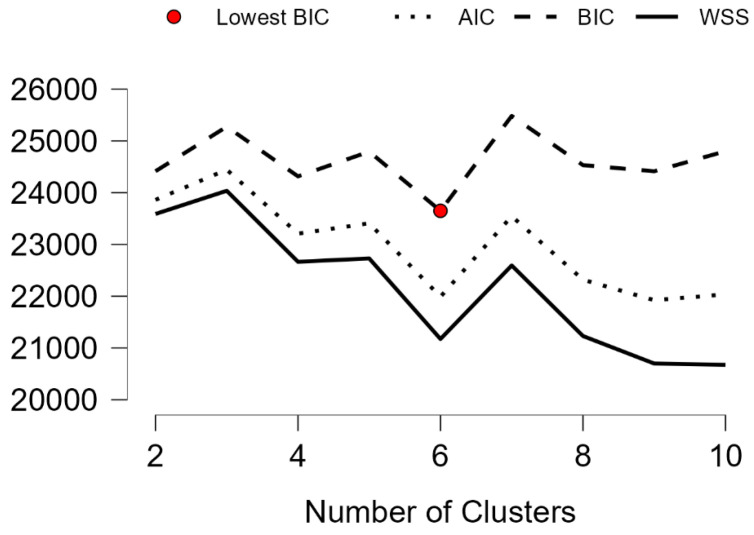
Elbow plot.

The elbow method generated a plot with the total within sum of squares on the y-axis and the number of clusters on the x-axis (Figure 1). The plot can be used for determining the optimal number of clusters, which in our case is six. The plot shows three curves using AIC, BIC, and elbow method optimization.

### 4.2. Exploratory Factor Analysis Results

The factorability of the 68 BCPS items was first investigated. We utilized a number of well-accepted criteria to determine if a relationship may be calculated. First, it was discovered that every single one of the 68 items showed a strong correlation with at least half of the other items, indicating plausible factorability. Second, the Bartlett’s test of sphericity was significant at 2, and the Kaiser–Meyer–Olkin measure of sample adequacy was 0.713, which is above the generally advised threshold of 6, χ^2^ (2278) = 16,787.285, *p* < 0.001. The Chi-squared test was also significant at 4065.240 (1885), *p* < 0.001.

Finally, as seen in Table 3, the communalities were all over 0.407, further demonstrating that each item shared some variation with other items. These broad indications led to the conclusion that factor analysis was appropriate for all 68 items.

Because the main objective was to identify and compute composite scores for the factors underlying the 68-item BCPS version, principal components analysis was performed.

The initial eigenvalues indicated that the six factors’ unrotated and rotated solution explained a cumulative 45% of the variance, with the first factor explaining 30%, the second factor explaining 4%, the third factor explaining 3%, the fourth factor explaining 2%, the fifth factor explaining 2%, and the sixth factor explaining 1%.

**Figure 2:**
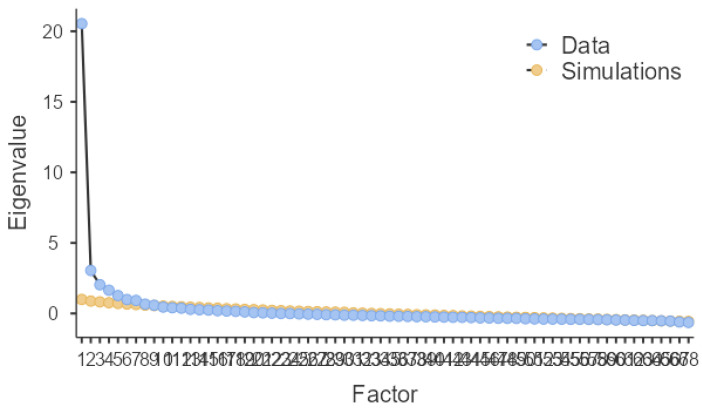
Scree plot.

Due to the leveling out of eigenvalues on the scree plot (Figure 2) after six factors, the six-factor solution, which explained 45% of the variation, was chosen for the statistical pre-evaluation of the BCPS.

Additional fit indices yielded an RMSEA of 0.052 and a TLI of 0.816 for the entire 68-item BCPS, indicating an acceptable general model fit.

### 4.3. Network Analysis Results

The network structure of variables may be analyzed using network analysis. To ensure the correctness of our network analysis, the network needs to be a sufficiently accurate representation of the underlying data in order to guarantee the scientific accuracy [61]. Since the aim of this study is to bring as much as possible statistical evidence for the six-factorial structure of the Bean Counter Profiling Scale, we have employed network analysis for adding empirical rigor [62,63,64,65] besides results obtained with factorial and cluster analysis. 

A network is a collection of structures that include variables represented by nodes and the links (officially termed edges) between these nodes. Cross-sectional data from a group can indicate conditional independence connections at the group level [66].

Nodes represent items in psychological networks, whereas edges reflect correlations or predictive associations that may be calculated from data. In our case, a node represents a single item on a scale.

The direction and strength of the connection between nodes, or in our case, items, are indicated by edges. The edge may be positive, as in the case of positive covariance or correlation between the items, or it may be negative. Different colored lines to depict the edges of the graph show the polarity of the interactions: positive relationships are often colored blue or green, while negative relationships are typically colored red [67]. You can have weighted or unweighted edges. A weighted edge changes the thickness and color density of the edge linking the nodes to show the strength of a node-to-node link: larger, denser colored lines denote stronger relationships. In a network where there are no connections between nodes, the edge may instead be unweighted and merely indicate whether a link is present or absent.

We have processed our data using R-packages: bootnet, glasso, huge and mgm [68,69,70,71,72,73,74,75,76,77,78,79].

To keep the graph as clean as possible, thus reducing the noise of the items that were previously discovered as not fitting the six-factor structure, we have eliminated items that did not load on any factor in the exploratory factor analysis. Thus, our network analysis investigated the relationship between 46 items of the total of 68 items of BCPS.

This network has 46 nodes, a maximum of 1035 edges, and a sparsity value of 0.719. The number of edges estimated was decreased to 291 by the analysis’s usage of the EBICglasso estimation.

**Figure 3:**
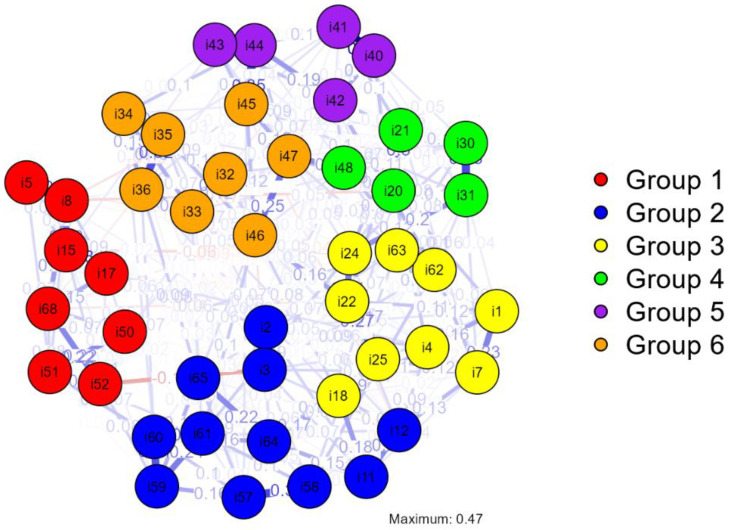
Network of the 46 items of BCPS.

Figure 3 shows a visualization of the network of the 46 items of BCPS, which are depicted by six different groups colored in different colors, as seen in the legend. For example, items 5, 8, 15, 17, 50, 51, 52 and 68 belong to group 1 depicted in red color. As the purpose of this research is just the pre-validation of the BCPS, we will not detail the theoretical meaning of the groupings; we are only delineating the scale network structure.

As shown in Figure 3, nodes are associated both positively and negatively with one another.

As seen in Table 4, there are four centrality measures employed: betweenness, closeness, strength and expected influence to identify highly influential nodes [80].

According to the centrality of closeness, a node’s proximity to every other node in the network is measured. It is calculated as the mean of the shortest paths connecting each network node. The total distance between a node and all other nodes is inversely proportional to a node’s centrality. One technique for understanding closeness is as a metric of how long it will take for information to spread sequentially from one node to every other node. Betweenness centrality counts how frequently a node is on the shortest route between other nodes. Betweenness centrality is frequently used to quantify a node’s dependence on other nodes and, thus, its potential for control. A node’s influence on its immediate neighbors or nodes with which it has an edge is determined by its strength, which is the sum of all the absolute values of connections with other nodes in the network [80].

Item 46 has the biggest effect over the flow between all items in terms of betweenness. In terms of closeness, the item best placed to influence the entire network most quickly is the same item 46. In terms of strength, the most influential item over its immediate neighbors is item 44, and in terms of expected influence, the same item 44 presents the most prominent characteristics in the analyzed network.

## 5. Discussion

There have been no published experimental investigations that use fuzzy c-means in the pre-validation of profiling scales; hence, our technique is completely novel, especially when our design process is rooted in an exploratory investigation of psychological factors accounting for the bean counter profile. With this original factor–cluster–network analysis of the 68-item Bean Counter Profiling Scale, we have statistically prototyped the six-dimensionality structure.

As results clearly pointed out, the six-dimension scale yielded by all techniques involved in this pre-validation study—fuzzy c-means unsupervised hierarchical clustering algorithm, exploratory factor analysis and network analysis. We found strong evidence for further qualitatively investigating the scale dimensionality and depicting the theoretical explanation behind each of the six dimensions. We anticipate a high correlation with all the big five personality dimensions: openness, conscientiousness, extraversion, agreeableness, and neuroticism, as all items extracted from the literature depict both general and very specific behaviors of accountants, mostly in terms of personality characteristics.

## 6. Conclusions

Over the last few years, the need for a systematic and comprehensive approach to cognitive concerns in the design of sociotechnical systems has evolved as computer-based technologies have pushed the nature of operational work in a path where cognitive challenges predominate [81,82,83,84].

Decision making in complex and dynamic information settings, remote cooperation, and the administration of substantially networked systems have all revolutionized the nature of work in many circumstances. Cognitive systems engineers discover the cognitive states, cognitive processes, and cognitive strategies utilized by competent practitioners to conduct this task and then provide design solutions for tools that enable expert human cognition, such as decision and planning tools.

Human cognition is one critical dimension on which sociotechnical systems can fail, often catastrophically. On the other hand, it is a dimension that has the potential to significantly improve the overall performance of a sociotechnical system. Cognitive system engineers, in particular, do not regard the human as a user or operator but rather as an entity having functional features that contribute to system performance.

For determining user requirements and creating acceptable and effective computer-based information aid systems, traditional human–computer interaction (HCI) and system design paradigms have proven ineffective. By merging modeling aspects from engineering, psychology, cognitive science, information science, and computer science, cognitive systems engineering (CSE) offers a much wider, more dynamic framework. This study is one of the first practical applications of the burgeoning new subject of cognitive systems engineering in the construction of psychological resilient scales.

Distinct characteristics of a psychological construct are represented by different individual items, since they cannot be directly assessed. The underlying assumption in instrument development is that the concept is what motivates respondents to react similarly to all of these items. As a result, it is appropriate to combine the replies to all of these items into a single score and draw conclusions about the construct. Instruments for measuring can be used to measure a single construct, numerous separate constructions, or even smaller differences within a construct.

Even though a set of items appears to measure the same construct theoretically, the researcher must empirically demonstrate that the population studied exhibits a coherent response pattern throughout the set of items to support its use to measure the construct. If respondents do not react coherently, it shows that the items are not working as intended and may not all measure the same concept. As a result, the single number chosen to represent the build from that set of objects would reveal very little about the intended construct.

A multidimensional scale is an instrument designed to assess multiple related constructs or several separate facets of a construct. To be able to divide the findings into subscales, the items must measure distinctively different constructs. It is vital to remember that whether a group of objects reflects distinct constructions depends on the intended population, in this case accountants.

The coefficient alpha does not indicate whether the instrument assesses one or several underlying components [85,86]. In conclusion, publishing simply coefficient alpha, in our instance an overall Cronbach’s coefficient of 0.947, is insufficient evidence to make reliable interpretations of instrument scores or to demonstrate that a group of questions measures only one underlying concept, unidimensionality.

In order to determine whether participant responses to particular subsets of survey items are more closely related to one another than to other subsets, a statistical technique known as factor analysis examines the relationships between the survey items. In other words, it examines the dimensionality of the survey items [87,88,89]. This method was specifically created to clarify the dimensionality that underlies sets of achievement test items [90]. In terms of constructs, factor analysis may be used to investigate the likelihood that a certain collection of items collectively measures a predetermined construct, hence obtaining validity data on internal architecture.

As a result, EFA can clarify the connections between various concepts and structures and contribute to the creation of new frameworks. Early on in the creation of an instrument, EFA is appropriate. The researcher can identify survey questions that do not empirically fit the desired construct and should be eliminated by employing EFA. EFA can also be used to investigate the instrument’s dimensionality. In EFA, it is considered that the variance that the items share represents the concept and that the nonshared variance represents measurement flaws.

In terms of mathematics, factor analysis examines the variances and covariances among the components. It is believed that the construct is represented by the common variance among the components. The constructs are frequently referred to as factors in factor analysis. Error variance is referred to as nonshared variance. The covariances among all items are examined collectively during an EFA, and items that share a significant amount of variance are collapsed into a factor. A CFA extracts the common variation across items that are designed in advance to assess the same underlying concept. In EFA, it is not required to make an a priori assumption about which items correspond to which factors because the EFA establishes these associations. Before applying the measuring instrument for study, researchers should preferably corroborate the six-factorial structure we established using EFA with a CFA on diverse accountant populations.

All of the strategies described give viable answers that may be examined to determine the optimal solution. When these indices agree, it indicates that the data have a distinct factor structure. It is critical that the number and character of factors make theoretical sense in order for each component to be interpretable. Furthermore, the scale’s intended usage should be evaluated.

The proposed six-factor approach necessitates additional refinement, which is the primary limitation of this pre-validation research. Another disadvantage of this study is that the sample was 70% female, which is an over-representation of women in the accounting community. More research should be conducted to see if the stated dimensionality holds true in a larger sample. Future study should look at whether the instrument has the same structure when used with accountants from various backgrounds. The work given here merely establishes the BCPS’s dimensionality. We advocate gathering additional forms of validity data, such as evidence based on content or correlations to other factors, to improve our confidence that the scores from this scale accurately represent the bean counter personality profile.

## Figures and Tables

**Table 1:** Main characteristics of Factor-Cluster-Network Anaysis.

Factor Analysis	Cluster Analysis	Network Analysis
Dimension reduction technique	Classification analysis	Measures the fit of the clustering to the network data
A method for simplification	A method for categorization	A method for grouping items in a scale into different classes based on their links and identify relation among classes
Summarizes inter-related items into latent constructs	Classifies items showing the same characteristics into clusters	Groups items into classes based on their links as well as their attributes
The objective is to explain correlation in a set of items and relate items to each other	The objective is to address heterogeneity in each set of items	Operating at multiple levels, it describes and makes inferences about relational properties of items, dimensions, and of entire scale
Number of items reduction	Number of observation reduction	Number of non-zero edges reduction

**Table 2:** Fuzzy c-means clustering information.

Cluster	1	2	3	4	5	6
Size	11	10	262	3	130	16
Explained proportion within-cluster heterogeneity	0.017	0.027	0.708	0.006	0.192	0.050
Within sum of squares	423.931	657.042	17,400.350	157.315	4713.965	1238.947
Silhouette score	0.013	0.051	−0.027	0.005	0.101	−0.087
Center i1	0.766	0.764	−0.555	0.784	0.739	0.758
Center i2	0.475	0.470	0.420	0.484	0.458	0.469
Center i3	0.437	0.430	0.385	0.445	0.423	0.433
Center i4	0.877	0.881	−0.392	0.903	−0.378	0.872
Center i5	−1.212	−1.228	0.850	−1.256	−1.159	−1.210
Center i6	−0.308	0.728	0.642	0.748	0.710	0.721
Center i7	0.626	0.620	0.544	0.638	0.608	0.618
Center i8	−0.916	−0.927	1.106	−0.949	−0.885	−0.915
Center i9	0.661	0.654	−0.649	0.674	0.640	0.653
Center i10	0.770	0.766	−0.241	−3.503	−0.233	−1.304
Center i11	0.644	−0.673	0.567	−4.693	−0.599	0.637
Center i12	−0.538	0.642	0.571	0.660	0.626	0.639
Center i13	−0.444	0.702	0.625	0.720	0.688	−1.612
Center i14	−0.559	0.667	−0.529	0.686	0.653	0.664
Center i15	0.628	2.067	1.241	−0.801	−0.746	−0.775
Center i16	0.982	0.986	0.084	−0.800	0.948	0.972
Center i17	0.161	−0.508	0.781	−1.207	0.772	−1.165
Center i18	−0.445	−0.474	−0.427	0.776	0.735	0.752
Center i19	0.039	0.949	0.018	−0.923	0.054	0.024
Center i20	0.688	0.684	−0.542	0.703	0.670	0.678
Center i21	0.836	0.832	−0.237	0.855	0.812	−1.311
Center i22	0.997	−0.181	−0.175	1.021	0.962	0.984
Center i23	0.763	−0.469	−0.434	0.777	0.740	0.750
Center i24	0.802	−0.482	−0.452	0.817	0.778	0.790
Center i25	0.820	0.814	−0.383	0.838	−0.360	0.810
Center i26	0.848	0.854	0.112	−2.133	0.125	0.844
Center i27	−0.429	−0.455	−0.417	−1.689	0.731	0.739
Center i28	1.036	1.033	−0.076	−1.215	−0.036	−0.074
Center i29	−0.677	0.664	−0.645	0.685	0.654	0.663
Center i30	−0.539	0.719	−0.517	−1.886	0.705	−1.813
Center i31	−1.857	0.757	−0.530	0.780	0.742	0.752
Center i32	−0.627	0.282	0.258	−0.666	0.293	−0.642
Center i33	0.975	−0.001	0.001	−0.986	0.942	−0.948
Center i34	0.897	−0.013	−0.008	0.915	0.866	−0.007
Center i35	0.279	−0.658	0.241	0.271	0.277	−0.642
Center i36	0.162	−0.795	0.130	−0.808	0.168	0.143
Center i37	0.573	0.564	0.493	0.583	−0.783	0.564
Center i38	0.701	−0.610	−0.554	0.714	−0.535	0.691
Center i39	0.734	−0.624	0.640	0.754	−0.551	0.728
Center i40	−1.142	−0.213	0.675	0.777	0.739	0.748
Center i41	0.004	−0.908	−0.012	0.903	0.857	−0.010
Center i42	0.661	−1.480	−0.367	0.677	0.644	−1.450
Center i43	1.162	1.166	0.339	−0.410	0.383	−0.398
Center i44	0.918	0.068	0.809	0.074	0.888	−0.763
Center i45	0.882	0.878	0.036	0.901	0.076	−0.770
Center i46	0.963	−0.009	−0.009	−0.003	−0.871	−0.952
Center i47	0.779	0.770	−0.319	0.793	0.754	0.762
Center i48	0.678	0.671	0.592	0.691	0.657	0.666
Center i49	−3.395	0.376	0.346	0.388	0.366	0.378
Center i50	−1.802	−1.838	0.264	1.002	−1.057	0.964
Center i51	0.120	−1.123	0.688	0.122	−0.467	−1.101
Center i52	−0.642	−0.652	1.398	−0.665	−0.614	−0.645
Center i53	0.766	0.115	0.718	−1.221	−1.121	1.408
Center i54	−0.197	−1.251	−0.195	0.842	0.790	−1.222
Center i55	−1.272	−1.315	0.299	−0.502	0.329	0.321
Center i56	0.047	0.886	0.025	−0.845	0.863	−0.812
Center i57	0.023	−0.801	0.008	0.006	−0.713	−0.779
Center i58	0.542	−1.603	−0.472	0.553	0.523	0.534
Center i59	0.830	−1.705	−0.000	−0.874	0.013	−0.842
Center i60	0.915	−1.754	0.033	−0.878	0.053	−0.843
Center i61	0.745	−1.177	−0.189	0.757	0.721	−1.148
Center i62	0.666	−2.134	−0.665	0.681	0.648	0.658
Center i63	−0.493	−1.737	−0.465	−1.765	−0.449	0.691
Center i64	0.787	−1.393	−0.265	0.804	−1.269	0.779
Center i65	0.001	−1.054	−0.013	−1.067	0.012	−1.025
Center i66	−0.283	0.502	0.460	−0.301	0.487	−0.291
Center i67	0.918	−1.184	−0.114	0.938	−1.074	0.907
Center i68	−1.073	−1.085	0.996	−1.107	0.993	−1.067

*Note.* The between sum of squares of the 6-cluster model is 7870.66. The total sum of squares of the 6-cluster model is 32,462.21.

**Table 3:** Factor Loadings.

Items	Factor 1	Factor 2	Factor 3	Factor 4	Factor 5	Factor 6	Uniqueness
i44	0.771						0.331
i43	0.696						0.509
i36	0.640						0.344
i35	0.616						0.380
i45	0.615						0.416
i41	0.570						0.506
i34	0.561						0.462
i33	0.495						0.492
i47	0.490						0.430
i42	0.472						0.538
i32	0.465						0.602
i40	0.463						0.577
i46	0.418						0.429
i48	0.407						0.586
i25		0.740					0.402
i24		0.705					0.322
i22		0.684					0.344
i18		0.596					0.451
i1		0.532					0.622
i20		0.523					0.470
i31		0.514					0.456
i7		0.504					0.533
i21		0.450					0.438
i30		0.410					0.549
i4		0.401					0.687
i60			0.753				0.334
i59			0.697				0.475
i61			0.697				0.392
i57			0.582				0.570
i65			0.549				0.519
i58			0.483				0.615
i64			0.447				0.477
i3				0.545			0.599
i2				0.502			0.656
i11				0.456			0.546
i12				0.418			0.529
i63					0.502		0.489
i62					0.489		0.507
i8						0.649	0.546
i17						0.617	0.588
i15						0.590	0.623
i68						0.547	0.641
i5						0.542	0.677
i52						0.466	0.652
i51						0.415	0.675
i50						0.408	0.746

**Table 4:** Centrality measures per variable.

	Network			
Variable	Betweenness	Closeness	Strength	Expected Influence
i44	1.427	0.506	2.151	2.435
i43	−1.190	−0.254	−1.032	−1.209
i36	−0.418	−0.474	0.889	0.807
i35	0.054	−0.273	0.476	0.850
i41	−0.761	−0.822	−0.152	0.256
i45	0.698	0.901	0.226	−0.742
i33	−0.590	−1.302	0.063	0.460
i34	−0.933	−0.997	−0.285	0.130
i47	0.612	1.429	1.224	1.559
i42	−0.761	−0.132	−0.798	−0.356
i32	−0.933	−1.280	−1.476	−0.997
i40	−0.547	−0.919	−0.403	0.019
i46	2.114	1.960	0.426	0.803
i48	−1.190	0.230	−0.772	−0.565
i24	0.998	1.512	2.089	0.912
i25	0.741	1.455	−0.047	0.355
i22	0.955	1.793	1.221	0.781
i18	0.355	0.854	0.618	0.985
i1	−1.147	−0.011	−1.348	−0.876
i20	1.814	1.221	1.002	−0.044
i31	−0.804	0.363	0.233	0.621
i7	0.355	0.563	0.143	0.535
i21	0.612	0.842	0.232	0.619
i30	−1.276	−0.299	−0.451	−0.027
i4	−0.976	0.165	−1.460	−0.982
i59	0.483	0.594	−0.082	0.322
i60	1.127	0.801	1.657	1.968
i61	0.483	0.844	0.932	1.282
i57	−0.547	−0.691	−0.553	−0.267
i65	−0.375	0.418	−0.586	−0.155
i58	−0.761	−0.934	−1.229	−0.763
i64	0.183	0.657	0.011	0.411
i2	0.183	0.166	0.229	0.502
i3	1.127	0.367	1.742	−0.731
i11	−1.147	−0.227	−0.344	0.074
i12	−1.147	−0.361	−0.197	−0.389
i62	1.985	1.056	0.402	0.565
i63	0.655	0.618	0.625	0.649
i8	1.513	−1.211	1.064	−0.446
i17	−0.933	−1.642	−0.075	0.151
i15	−0.633	−1.754	0.002	−0.999
i5	−1.662	−2.023	−1.863	−1.933
i68	0.312	−1.073	−0.843	−1.930
i50	−0.976	−1.353	−2.298	−1.776
i51	−0.332	−0.703	−1.035	−0.580
i52	1.256	−0.585	−0.330	−2.285

## Data Availability

Data will be made available on request by the first author and corresponding authors.
